# Supplementary material for: Structural basis for broad anti-phage immunity by DISARM
Source: Nat Commun. 2022 May 27;13:2987. doi: 10.1038/s41467-022-30673-1 (PMC9142583; doi:10.1038/s41467-022-30673-1)

Supplementary Information

Jack P. K. Bravo, Cristian Aparicio-Maldonado, Franklin L. Nobrega, Stan J. J. Brouns, and David W. Taylor

Supplementary Table 1 l Cryo-EM data collection, refinement, and validation statistics.

	**DrmAB-ADP** (PDB 7S9V, EMD-24938)	**DrmAB-ADP-DNA** (PDB 7S9W, EMD-24939)	**DrmAB**
**Data collection and processing**			
Magnification	45,000		
Voltage (kV)	300 kV		
Electron exposure (e–/Å^2)	80		
Defocus range (μm)	-1.5 to -2.5		
Pixel size (Å)	1.1		
Symmetry imposed	C1		
Initial particle images (no.)	4,669,932		1,540,419
Final particle images (no.)	120,119	121,764	148,062
Map resolution (Å) at 0.143 FSC threshold	3.31	3.41	3.84
Map resolution range (Å)	3.1 to 4.3	3.3 to 4.5	3.6-6.8
**Refinement**			
Initial model used (PDB code)	NA		
Model resolution (Å)	3.6	3.8	
FSC threshold	0.5		
Map sharpening *B* factor (Å^2)	-136.8	-151.1	
Model composition<br>Non-hydrogen atoms<br>Protein residues<br>Nucleotides<br>Ligands	 27680<br>1808<br>0<br>1	 28125<br>1751<br>7<br>1	
Mean *B* factors (Å^2)<br>Protein<br>Nucleotides	 62.08<br>N/A	 106.66<br>116.37	
R.m.s. deviations<br>Bond lengths (Å)<br>Bond angles (°)	 0.002<br>0.617	 0.002<br>0.631	
Validation<br>MolProbity score<br>Clashscore<br>Poor rotamers (%)	 1.52<br>3.27<br>0	 1.53<br>3.34<br>0	
Ramachandran plot<br>Favored (%)<br>Allowed (%)<br>Disallowed (%)	 93.93<br>6.01<br>0.6	 93.90<br>6.1<br>0	

Supplementary Table 2 l DNA oligos used for *in vitro* assays in this study.

Oligo name	Sequence (5'-3')
DNA stem loop	TTTTTTTCATCGATGAGCACTGCTATTCCCTAGCAGTGCTCATC GATGATTTTCATCGATGAGCGGTTTT
Methylated DNA stem loop*	TTTTTTTCAT**mC**GATGAGCACTGCTATTCCCTAGCAGTGCTCAT **mC**GATGATTTTCAT**mC**GATGAGCGGTTTT
Fluorescent DNA	Cy5-ATTTTGACAGCCCACATGGCTTGATGAGTGGCGCACTCGC CAGCCTGAGCATGGCGAAAACTCCTCCAGTCTGCT
Unlabeled complement DNA	AGCAGACTGGAGGAGTTTTCGCCATGCTCAGGCTGGCGAGTG CGCCACTCATCAAGCCATGTGGGCTGTCAAAAT
5' block	GCCATGTGGGCTGTCAAAAT
3' block	AGCAGACTGGAGGAGTTTTC

mC corresponds to 5-methyl-Cytosine.

Supplementary Table 3 l DNA oligos used for cloning in this study.

Oligo name	Sequence (5' - 3')	Used for: (in fw/rv couples)	fw/rv
BN1750	TACTTCCAATCCAATGCAATGATCAT CAATAACAAAACTCCAG	Clone *drmB* into plasmid 13SS using LIC cloning	fw
BN1751	TTATCCACTTCCAATGTTATTATTAAA AACCCTTAAAAAATGCAGC	Clone *drmB* into plasmid 13SS using LIC cloning	rv
BN1060	TATATATACATATGACTGATAACAACA AATCTAG	Clone *drmA* into plasmid pACYC-Duet using RE cloning-NdeI	fw
BN1061	ATATATATCTCGAGTCAATCCTCGTC TTTCGTTG	Clone *drmA* into plasmid pACYC-Duet using RE cloning-XhoI	rv
BN172	AGATCTGCCATATGTATATCTCCTTC	Amplify pACYC-Duet backbone for cloning of *drmA* insert	rv
BN2290	GATCGCTGACGTCGGTACCCTCGAG TC	Amplify pACYC-Duet backbone for cloning of *drmA* insert	fw
BN1064	TATATATACATATGATGGATGAACTC TTAGATGC	Clone *drmC* into plasmid pET-Duet using RE cloning-NdeI	fw
BN1065	ATATATATCTCGAGTTAAATCAGACT AATGATATTTGTG	Clone *drmC* into plasmid pET-Duet using RE cloning-XhoI	rv
BN172	AGATCTGCCATATGTATATCTCCTTC	Amplify pET-Duet backbone for cloning of *drmC* insert	rv
BN2290	GATCGCTGACGTCGGTACCCTCGAG TC	Amplify pET-Duet backbone for cloning of *drmC* insert	fw
BN3096	CATACCCTATCTCATTTGTTGAT	Create mutant DrmB-ΔDUF1998 in 13ss and in pCDF	fw
BN3095	TTATCATTACAGCATGGCATATCGGA TAC	Create mutant DrmB-ΔDUF1998 in 13ss and in pCDF	rv
BN3104	TTATCATTATCGCATTGACATAGGTA CAG	Create mutant *drmA*-ΔPA in pCDF	rv
BN3105	GAAGTTGAGTCTGGTGTAC	Create mutant *drmA*-ΔPA in pCDF	fw
BN3436	TTGGTGCCCAGTAGCCTC	Create mutant *drmA*-Δloop in pACYC and pCDF	rv
BN3437	TTCGGCTGCTTCACGTGG	Create mutant *drmA*-Δloop in pACYC and pCDF	fw
BN3377	CAACACGACTGGTAGCCTGAATATAC	Amplify DNA from pCDF-Duet with *drmA*-ΔPA and clone into pACYC-Duet	rv
BN3378	GGACACCAAATTTATCAAGGACGATC	Amplify DNA from pCDF-Duet with *drmA*-ΔPA and clone into pACYC-Duet	fw
BN3375	GTATATTCAGGCTACCAGTCGTGTTG	Amplify DNA from synthetic block to clone in 13SS and pCDF-Duet for *drmA* mutants R1294A, V1296G, V1296W	fw
BN3376	GATCGTCCTTGATAAATTTGGTGTCC	Amplify DNA from synthetic block to clone in 13SS and pCDF-Duet for *drmA* mutants R1294A, V1296G, V1296W	rv
BN3377	CAACACGACTGGTAGCCTGAATATAC	Amplify vectors 13SS and pCDF-Duet DNA to insert amplified DNA of *drmA* mutants R1294A, V1296G, V1296W	rv
BN3378	GGACACCAAATTTATCAAGGACGATC	Amplify vectors 13SS and pCDF-Duet DNA to insert amplified DNA *drmA* mutants R1294A, V1296G, V1296W	fw

BN3379	GATGAACAGAACTTGAATGTGAGC	Amplify DNA from synthetic block to clone in 13SS and pCDF-Duet for *drmA* mutants R569D, Q572D, K610D, R658D, R649A, K803A, R810A	rv
BN3380	GATAATTAAATCAGGCGGTCGGATA G	Amplify DNA from synthetic block to clone in 13SS and pCDF-Duet for *drmA* mutants R569D, Q572D, K610D, R658D, R649A, K803A, R810A	fw
BN3381	GCTCACATTCAAGTTCTGTTCATC	Amplify vectors 13SS and pCDF-Duet DNA to insert amplified DNA *drmA* mutants R569D, Q572D, K610D, R658D, R649A, K803A, R810A	rv
BN3382	CTATCCGACCGCCTGATTTAATTATC	Amplify vectors 13SS and pCDF-Duet DNA to insert amplified DNA *drmA* mutants R569D, Q572D, K610D, R658D, R649A, K803A, R810A	fw

Supplementary Table 4 l Plasmids used in this study.

Plasmid	Description	Name in paper	Antibiotic resistance marker	Source
pTU515	13SS-*drmB* fused to His-tag		Spectinomycin	This paper
pTU518	13SS-*drmB*-ΔDUF1998 fused to His-tag		Spectinomycin	This paper
pTU516	pACYC-Duet-*drmA*		Chloramphenicol	This paper
pTU520	pACYC-Duet-*drmA*(ΔPA)		Chloramphenicol	This paper
pTU542	pACYC-Duet-*drmA*(Δloop)		Chloramphenicol	This paper
pTU522	pACYC-Duet-*drmA*(V1296G)		Chloramphenicol	This paper
pTU523	pACYC-Duet-*drmA*(V1296W)		Chloramphenicol	This paper
pTU524	pACYC-Duet-*drmA*(R569D)		Chloramphenicol	This paper
pTU525	pACYC-Duet-*drmA*(Q572D)		Chloramphenicol	This paper
pTU526	pACYC-Duet-*drmA*(K610D)		Chloramphenicol	This paper
pTU527	pACYC-Duet-*drmA(*R658D)		Chloramphenicol	This paper
pTU528	pACYC-Duet-*drmA*(R649A)		Chloramphenicol	This paper
pTU529	pACYC-Duet-*drmA*(K803A)		Chloramphenicol	This paper
pTU530	pACYC-Duet-*drmA*(R810A)		Chloramphenicol	This paper
pTU531	pACYC-Duet-*drmA*(R1294A)		Chloramphenicol	This paper
pTU517	pET-Duet-*drmC*		Ampicillin	Reference (28)
pTU495	pCDF-Duet-*drmABC*		Streptomycin	Reference (28)
pTU519	pCDF-Duet-*drmABC* with *drmB*-ΔDUF1998		Streptomycin	This paper
pTU521	pCDF-Duet-drmABC with *drmA*(ΔPA)		Streptomycin	This paper
pTU543	pCDF-Duet-drmABC with *drmA*(Δloop)		Streptomycin	This paper
pTU532	pCDF-Duet-drmABC with *drmA*(V1296G)		Streptomycin	This paper
pTU533	pCDF-Duet-drmABC with *drmA*(V1296W)		Streptomycin	This paper
pTU534	pCDF-Duet-drmABC with *drmA*(R569D)		Streptomycin	This paper

pTU535	pCDF-Duet-drmABC with *drmA*(Q572D)		Streptomycin	This paper
pTU536	pCDF-Duet-drmABC with *drmA*(K610D)		Streptomycin	This paper
pTU537	pCDF-Duet-drmABC with *drmA(*R658D)		Streptomycin	This paper
pTU538	pCDF-Duet-drmABC with *drmA*(R649A)		Streptomycin	This paper
pTU539	pCDF-Duet-drmABC with *drmA*(K803A)		Streptomycin	This paper
pTU540	pCDF-Duet-drmABC with *drmA*(R810A)		Streptomycin	This paper
pTU541	pCDF-Duet-drmABC with *drmA*(R1294A)		Streptomycin	This paper

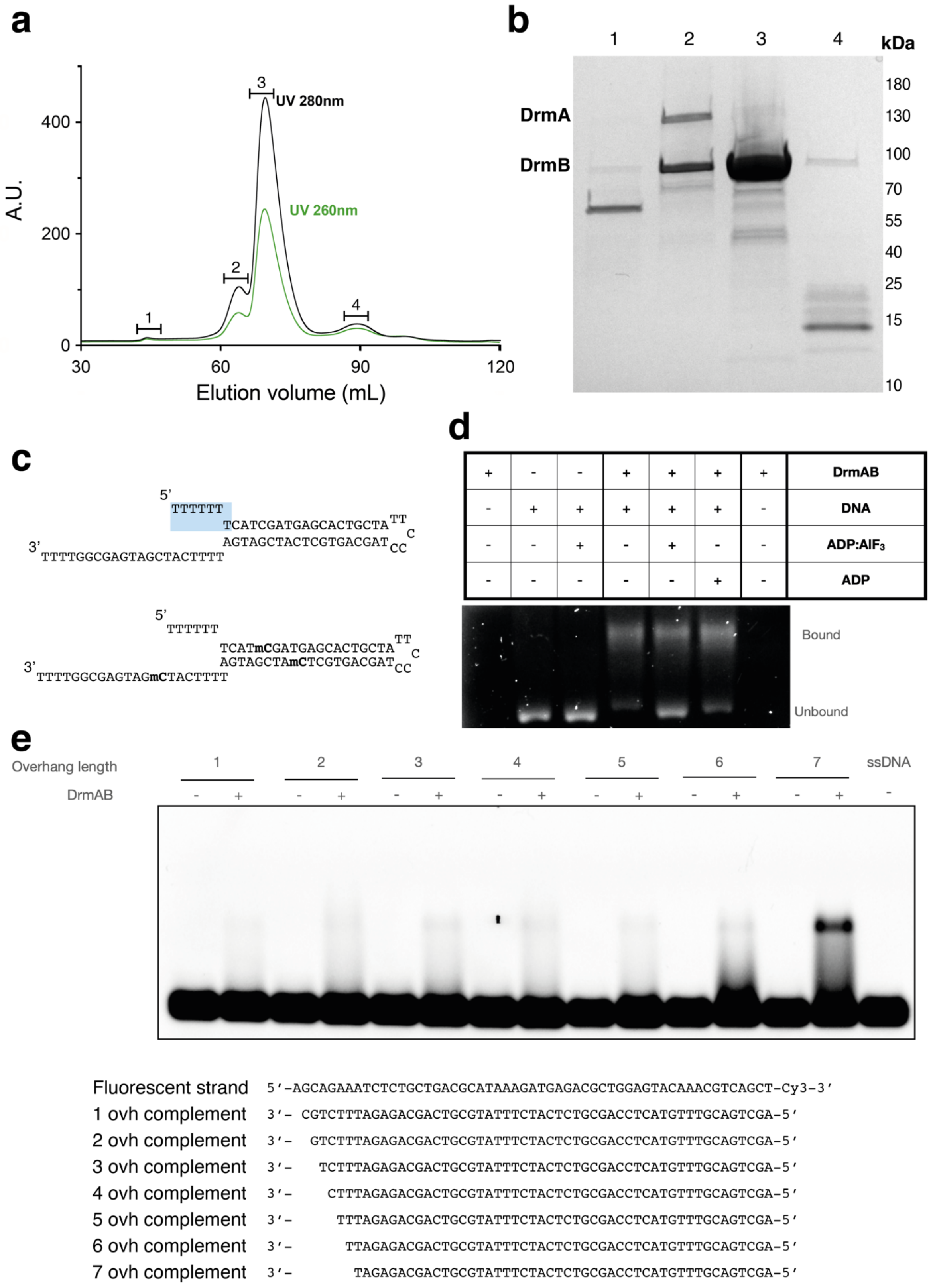
a
400
200
0
A.U.
30
60
90
120
Elution volume (mL)
UV 280nm
UV 260nm
1
2
3
4
b
1 2 3 4
kDa
DrmA
DrmB
180
130
100
70
55
40
25
15
10
c
5'
TTTTTT
TCATCGATGAGCACTGCTA TT C
AGTAGCTACTCGTGACGAT CC
3' TTTTGGCGAGTAGCTACTTTT
5'
TTTTTT
TCATmCGATGAGCACTGCTA TT C
AGTAGCTAmCTCGTGACGAT CC
3' TTTTGGCGAGTAGmCTACTTTT
d
DrmAB + - - + + + +
DNA - + + + + + -
ADP:AlF3 - - + - + - -
ADP - - - - - + -
Bound
Unbound
e
Overhang length 1 2 3 4 5 6 7 ssDNA
DrmAB - + - + - + - + - + - + - + -
Fluorescent strand 5'-AGCAGAAATCTCTGCTGACGCATAAAGATGAGACGCTGGAGTACAAACGTCAGCT-Cy3-3'
1 ovh complement 3'- CGTCTTTAGAGACGACTGCGTATTTCTACTCTGCGACCTCATGTTTGCAGTCGA-5'
2 ovh complement 3'- GTCTTTAGAGACGACTGCGTATTTCTACTCTGCGACCTCATGTTTGCAGTCGA-5'
3 ovh complement 3'- TCTTTAGAGACGACTGCGTATTTCTACTCTGCGACCTCATGTTTGCAGTCGA-5'
4 ovh complement 3'- CTTTAGAGACGACTGCGTATTTCTACTCTGCGACCTCATGTTTGCAGTCGA-5'
5 ovh complement 3'- TTTAGAGACGACTGCGTATTTCTACTCTGCGACCTCATGTTTGCAGTCGA-5'
6 ovh complement 3'- TTAGAGACGACTGCGTATTTCTACTCTGCGACCTCATGTTTGCAGTCGA-5'
7 ovh complement 3'- TAGAGACGACTGCGTATTTCTACTCTGCGACCTCATGTTTGCAGTCGA-5'

Supplementary Fig. 1 l DNA binding by DrmAB. a, Size-exclusion chromatogram of DrmAB complex. DrmB was tagged and used to pull down co-expressed DrmAB complex. Fractions 1 – 4 are analyzed in panel B. **b,** SDS PAGE analysis of DrmAB complex purified by size-exclusion chromatography. DrmAB assembles with a 1:1 DrmA:DrmB stoichiometry (fraction 2). Fraction 2 was used for subsequent structural and biochemical analysis. Representative of three independent experiments. Uncropped gel image is provided in **Supplementary Source Data 1**. **c**, Top: Secondary structure diagram of DNA stem-loop used for structural analyses (**Supplementary Table 2**). 5' region visible in cryo-EM reconstruction is denoted by orange box. Bottom: methylated DNA substrate used in ATPase assay **d**, Native gel shift assay used to determine sample preparation conditions for cryo-EM. 4 μM DNA hairpin (**Supplementary Table 2**) was heat annealed and incubated with 10 μM DrmAB in the absence or presence of 1 mM ADP or ADP:AlF_3. Bound and free DNA were separated using EMSA. Representative of three independent experiments. Uncropped gel image is provided in **Supplementary Source Data 2**. **e**, EMSA of binding Cy5-labelled DNA with increasing overhang length by annealing complementary DNA of decreasing lengths. Binding is first observed when a length of 7-nt is present. Substrates used in successive overhang binding assay are shown below. Representative of three independent experiments. Uncropped gel image is provided in **Supplementary Source Data 3**.

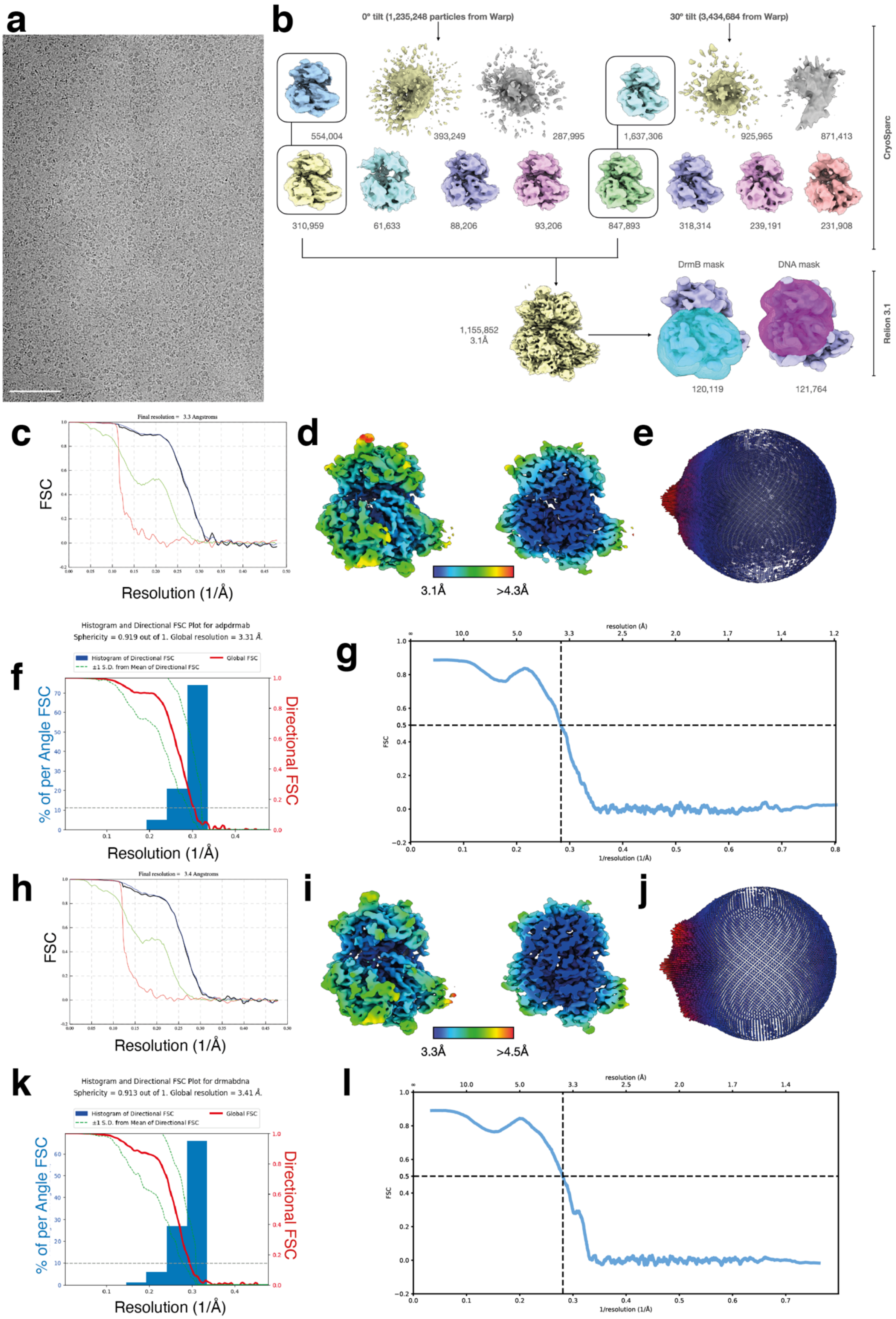
a
b
0° tilt (1,235,248 particles from Warp)
30° tilt (3,434,684 from Warp)
554,004
393,249
287,995
1,637,306
925,965
871,413
310,959
61,633
88,206
93,206
847,893
318,314
239,191
231,908
CryoSparc
DrmB mask
DNA mask
1,155,852
3.1Å
120,119
121,764
Relion 3.1
c
Final resolution = 3.3 Angstroms
FSC
Resolution (1/Å)
d
3.1Å
>4.3Å
e
Histogram and Directional FSC Plot for adpdrmab
Sphericity = 0.919 out of 1. Global resolution = 3.31 Å.
Histogram of Directional FSC
±1 S.D. from Mean of Directional FSC
Global FSC
f
% of per Angle FSC
Directional FSC
Resolution (1/Å)
g
resolution (Å)
FSC
1/resolution (1/Å)
h
Final resolution = 3.4 Angstroms
FSC
Resolution (1/Å)
i
3.3Å
>4.5Å
j
k
Histogram and Directional FSC Plot for drmabdna
Sphericity = 0.913 out of 1. Global resolution = 3.41 Å.
Histogram of Directional FSC
±1 S.D. from Mean of Directional FSC
Global FSC
% of per Angle FSC
Directional FSC
Resolution (1/Å)
l
resolution (Å)
FSC
1/resolution (1/Å)

Supplementary Fig. 2 l Cryo-EM data processing of DrmAB-ADP-DNA dataset. a, Representative cryo-electron micrograph of DrmAB:ADP:DNA. Scale bar – 100 nm. Representative of ~9,000 raw micrographs. **b,** Data processing workflow detailing how tilted and untilted particle stacks were combined to a consensus reconstruction. While further classification of said reconstruction yielded a 2.8Å-resolution map, the quality of density corresponding to both ssDNA and DrmB N-terminal half were poor. To ameliorate this, focused 3D classification using either a DrmB mask or a DNA mask was used. **c,** Gold-standard Fourier shell correlation (FSC) curve for DrmAB:ADP. **d,** EM density map of DrmAB:ADP color-coded according to local resolution. **e,** Angular distribution plot calculated in Relion of DrmAB:ADP. **F**: Directional 3D FSC for DrmAB:ADP calculated by 3DFSC. **g,** Map-to-model FSC for DrmAB:ADP. **h,** Gold-standard Fourier shell correlation (FSC) curve for DrmAB:ADP:DNA. **I:** EM density map of DrmAB:ADP:DNA color-coded according to local resolution. **j,** Angular distribution plot calculated in Relion of DrmAB:ADP:DNA. **k**, Directional 3D FSC for DrmAB:ADP:DNA calculated by 3DFSC (Zi Tan et al., 2017). **l**, Map-to-model FSC for DrmAB:ADP:DNA.

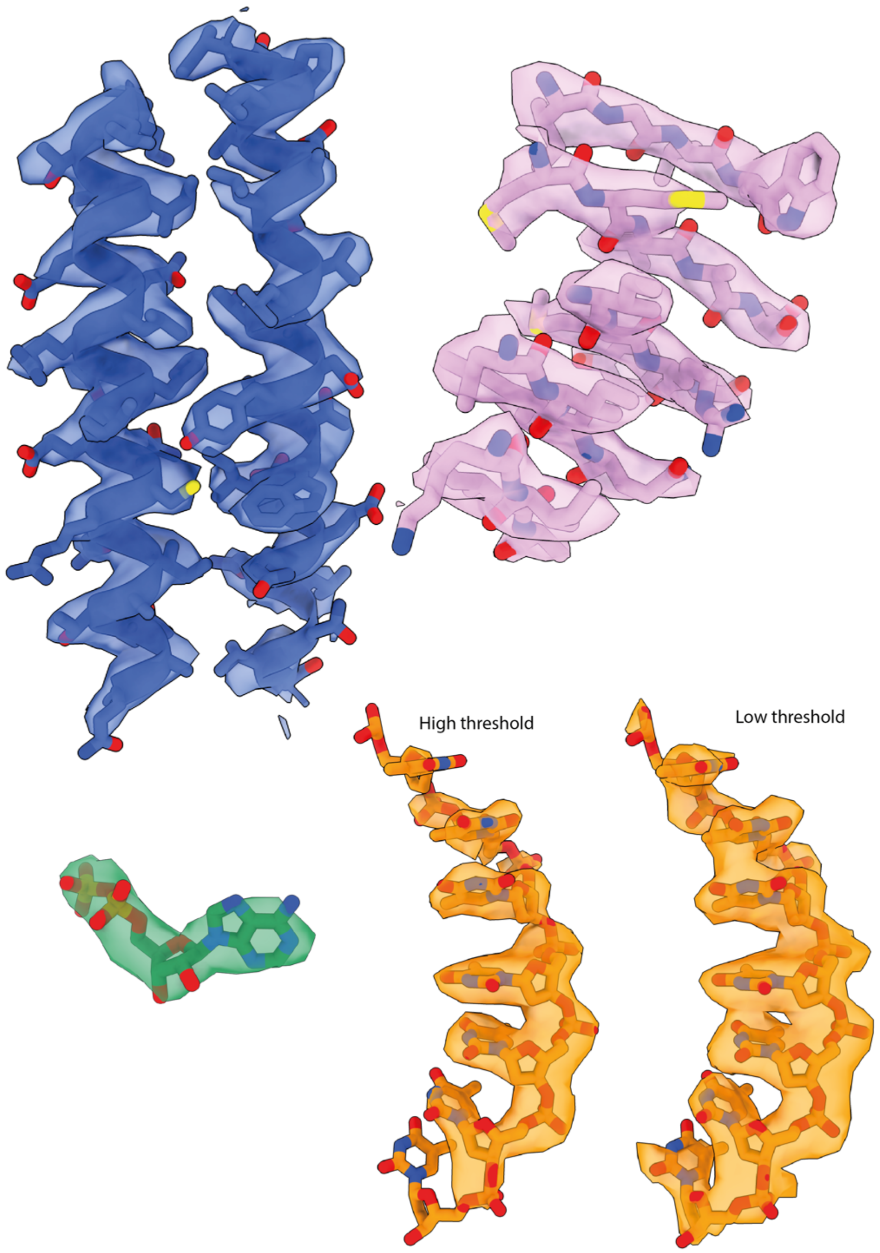

Supplementary Fig. 3 I Representative regions of DrmAB:ADP:DNA map with corresponding models. Blue: α-helices, pink: β-sheet, green: ADP, orange: DNA. DNA is shown at two different thresholds to illustrate that bases within the center of the molecule are well-resolved, but peripheral bases are less well-resolved, but can still be modelled with confidence.

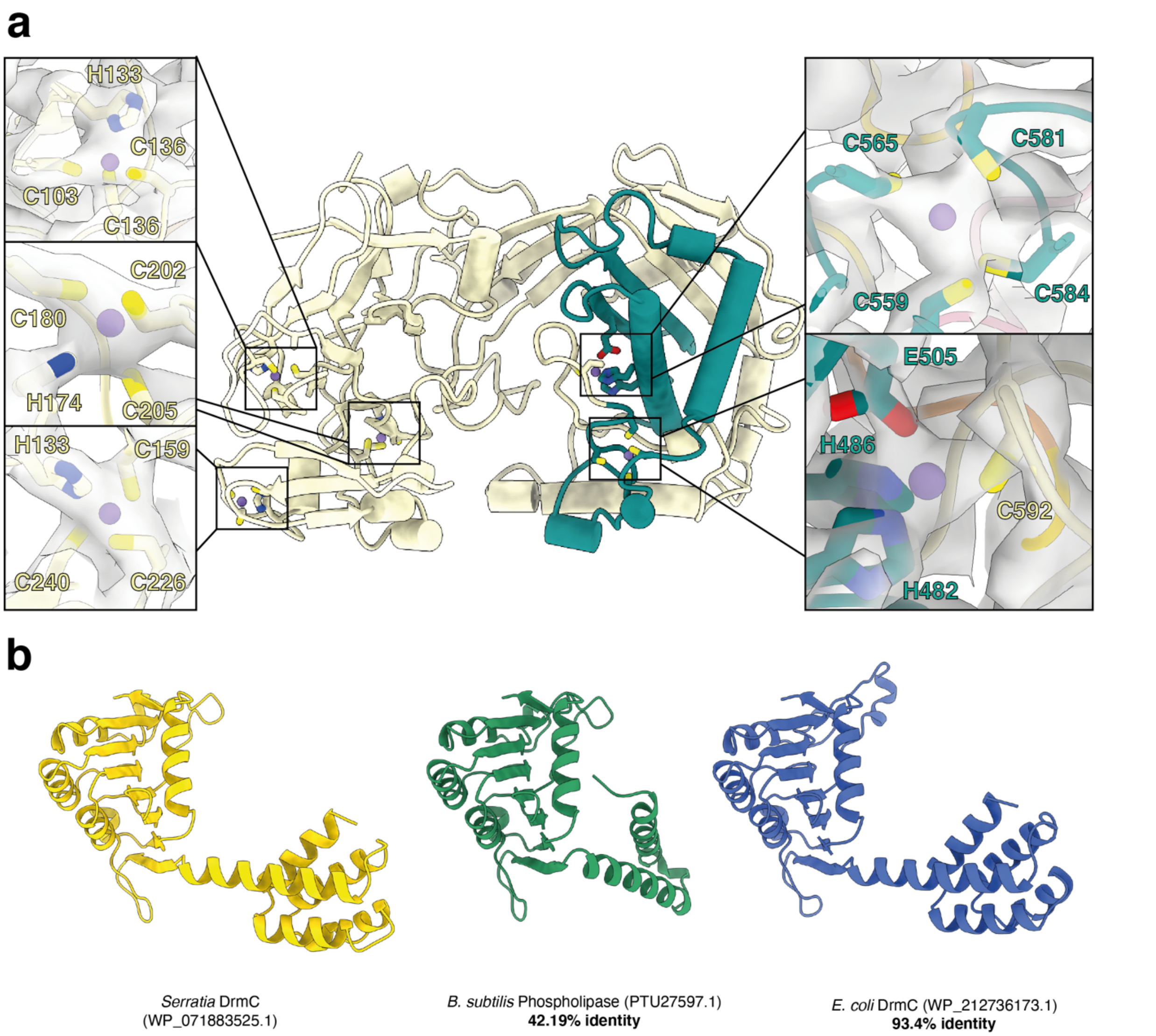

Supplementary Fig. 4 | Structural analysis of DrmB and DrmC. a, Multiple putative metal coordination sites within DrmB, with representative cryo-EM densities. **b,** Structural models of DrmC from *Serratia* (yellow), a *B. subtilis* PLD-nuclease protein homologue of DrmC (green) and an *E. coli* PLD-nuclease protein homologue of DrmC (blue). The DISARM operon in this study originated in *Serratia*, and *in vivo* assays were performed in *E. coli. B. subtilis* was the model organism used for *in vivo* assays in a previous DISARM study[7]. Structural models were generated using AlphaFold2.

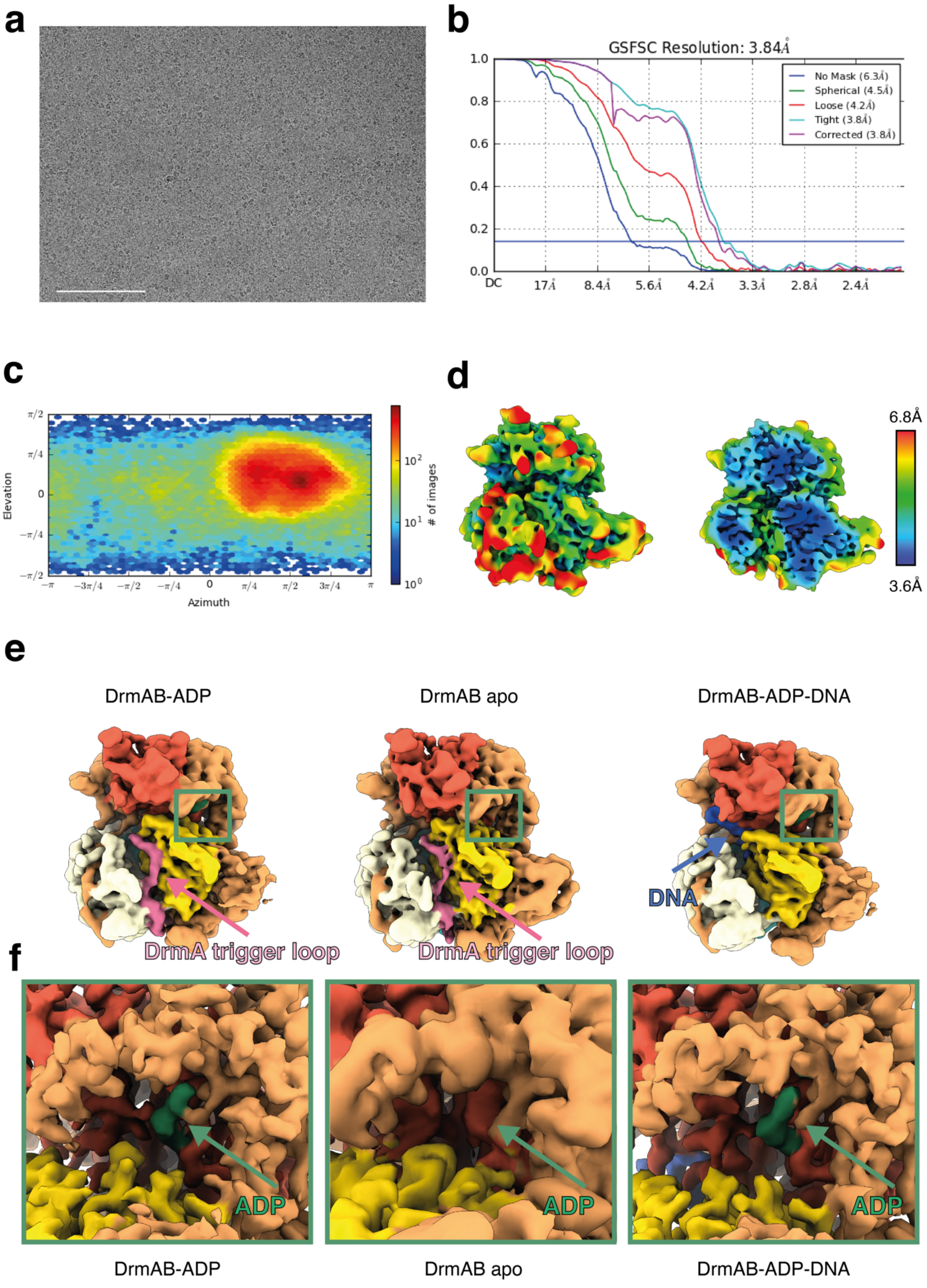
a
b
GSFSC Resolution: 3.84Å
No Mask (6.3Å)
Spherical (4.5Å)
Loose (4.2Å)
Tight (3.8Å)
Corrected (3.8Å)
1.0
0.8
0.6
0.4
0.2
0.0
DC
17Å
8.4Å
5.6Å
4.2Å
3.3Å
2.8Å
2.4Å
c
Elevation
Azimuth
# of images
d
6.8Å
3.6Å
e
DrmAB-ADP
DrmAB apo
DrmAB-ADP-DNA
DrmA trigger loop
DrmA trigger loop
DNA
f
ADP
ADP
ADP
DrmAB-ADP
DrmAB apo
DrmAB-ADP-DNA

Supplementary Fig. 5 I Structural features of DrmAB complex and TL. a, Representative cryo-electron micrograph of DrmAB. Scale bar – 100 nm. Representative of ~1,700 raw micrographs. **b**, Gold-standard FSC curves for DrmAB. **c**, Angular distribution plot for DrmAB calculated in cryoSPARC. **d,** EM density map of DrmAB color-coded according to local resolution. **e,** Presence of TL EM density features within DrmAB:ADP, DrmAB, and DrmAB:ADP:DNA maps. TL is pink, and DNA is blue. Green box corresponds to ATP binding site. **f,** ATP-binding site within DrmA RecA domains. DrmAB apo complex contains no nucleotide density, but still contains TL density.

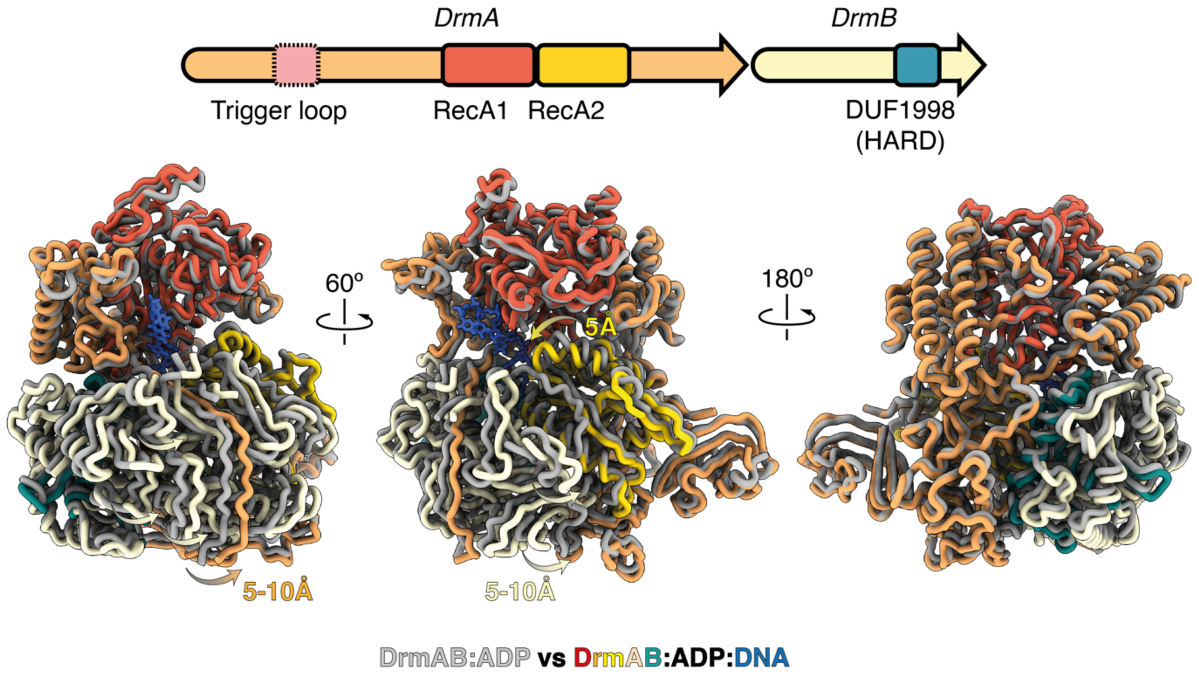

Supplementary Fig. 6 | Conformational changes upon DNA binding. Top, Domain architecture schematic of DrmA and DrmB. **Bottom**, DrmAB:ADP:DNA colored as in the above schematic, and the DrmAB:ADP complex colored in grey, showing conformational changes. DrmA trigger loop is absent in colored DrmAB:ADP:DNA model since it likely becomes disordered or flexible upon DNA binding.

Supplementary Source Data

Supplementary Source Data 1. Uncropped gel image for **Supplementary Fig. 1b**.

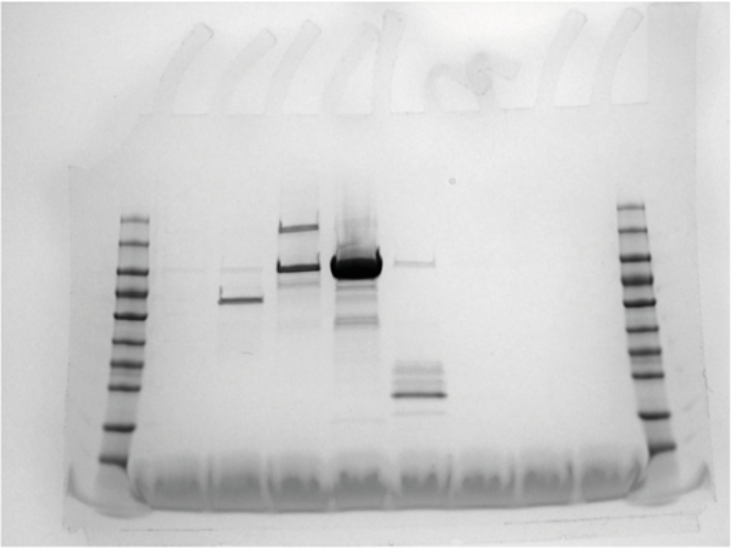

Supplementary Source Data 2. Uncropped gel image for **Supplementary Fig. 1d**.

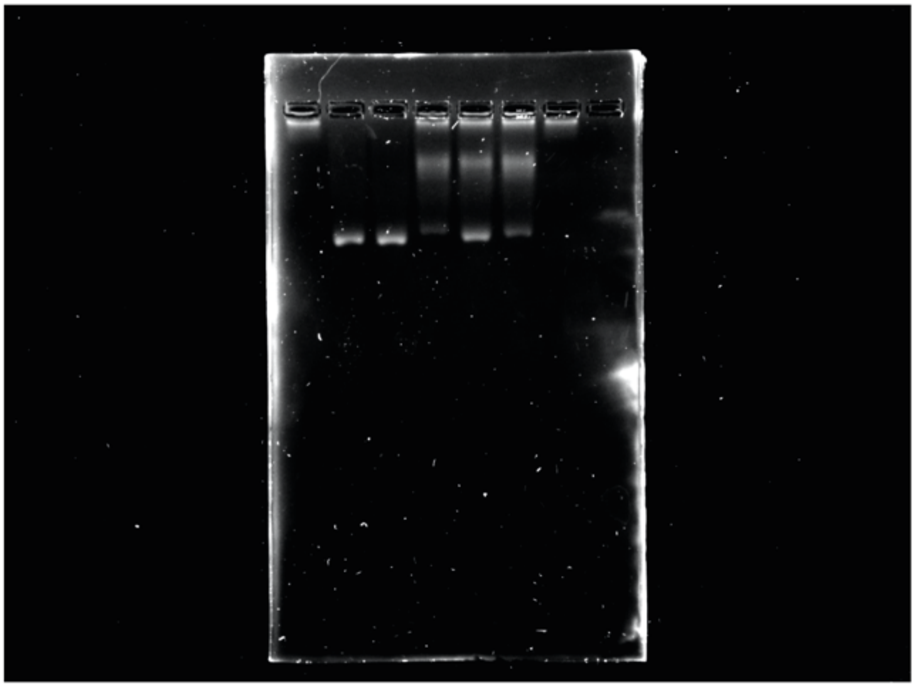

Supplementary Source Data 3. Uncropped gel image for **Supplementary Fig. 1e**.

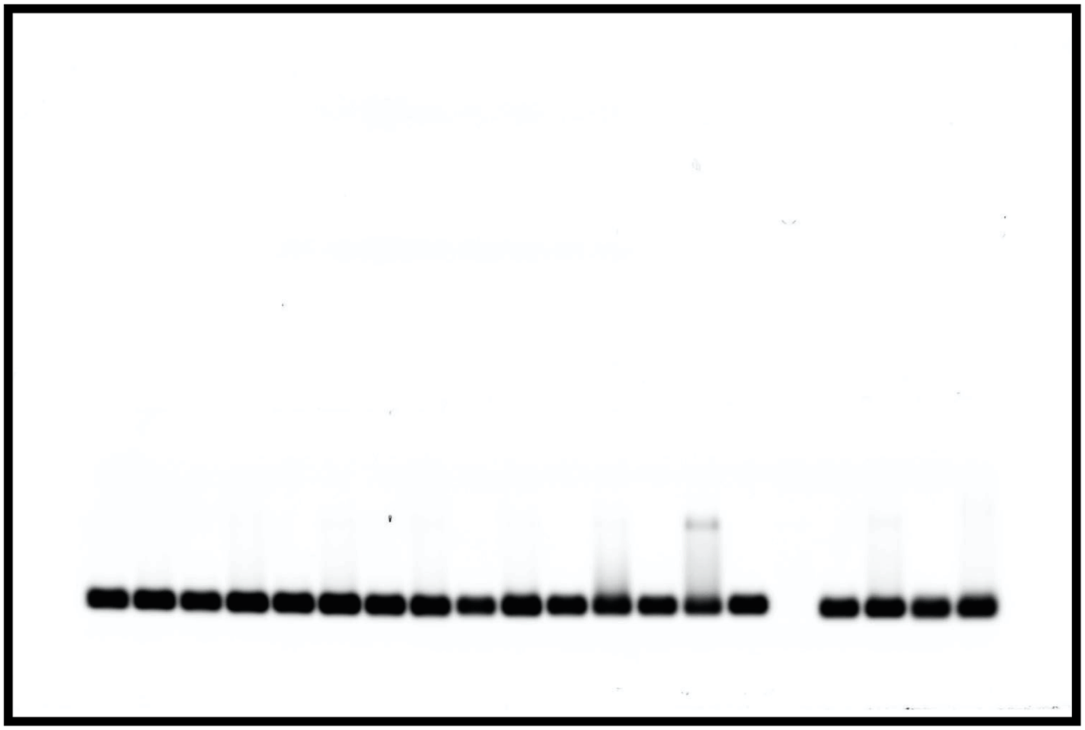

Supplement: Supplementary file 1 — Supplementary Information [file 41467_2022_30673_MOESM1_ESM.pdf]
